# The AUGIS Survival Predictor: Prediction of Long-Term and Conditional Survival After Esophagectomy Using Random Survival Forests

**DOI:** 10.1097/SLA.0000000000004794

**Published:** 2023-01-10

**Authors:** Saqib A. Rahman, Robert C. Walker, Nick Maynard, Nigel Trudgill, Tom Crosby, David A. Cromwell, Timothy J. Underwood

**Affiliations:** * School of Cancer Sciences, Faculty of Medicine, University of Southampton, Southampton, UK; † Oxford University Hospitals NHS Trust, UK; ‡ Sandwell and West Birmingham Hospitals NHS Trust, Birmingham, UK; § Velindre Cancer Center, Cardiff, UK; ¶ Clinical Effectiveness Unit, Royal College of Surgeons of England, London, UK

**Keywords:** esophageal cancer, esophagus, machine learning, prognostic model

## Abstract

The aim of this study was to develop a predictive model for overall survival after esophagectomy using pre/postoperative clinical data and machine learning.

**Summary Background Data:**

For patients with esophageal cancer, accurately predicting long-term survival after esophagectomy is challenging. This study investigated survival prediction after esophagectomy using a Random

Survival Forest (RSF) model derived from routine data from a large, well-curated, national dataset.

**Methods:**

Patients diagnosed with esophageal adenocarcinoma or squamous cell carcinoma between 2012 and 2018 in England and Wales who underwent an esophagectomy were included. Prediction models for overall survival were developed using the RSF method and Cox regression from 41 patient and disease characteristics. Calibration and discrimination (time-dependent area under the curve) were validated internally using bootstrap resampling.

**Results:**

The study analyzed 6399 patients, with 2625 deaths during follow-up. Median follow-up was 41 months. Overall survival was 47.1% at 5 years. The final RSF model included 14 variables and had excellent discrimination with a 5-year time-dependent area under the receiver operator curve of 83.9% [95% confidence interval (CI) 82.6%–84.9%], compared to 82.3% (95% CI 81.1%—83.3%) for the Cox model. The most important variables were lymph node involvement, pT stage, circumferential resection margin involvement (tumor at < 1 mm from cut edge) and age. There was a wide range of survival estimates even within TNM staging groups, with quintiles of prediction within Stage 3b ranging from 12.2% to 44.7% survival at 5 years.

**Conclusions:**

An RSF model for long-term survival after esophagectomy exhibited excellent discrimination and well-calibrated predictions. At a patient level, it provides more accuracy than TNM staging alone and could help in the delivery of tailored treatment and follow-up.

Esophagectomy for cancer is a highly morbid operation from which patients frequently take >18 months to recover.^1^–^3^ Long-term prognosis for patients also remains poor, with 5-year survival estimated to be <50%.^4^

Presently, clinicians have a limited number of tools to identify patients with esophageal cancer who are likely to respond well to surgery and those who may not. TNM staging is widely used for patient stratification, but the classification is based on largely historic data (patients treated in 1980 s—2000 s).^5^ In addition, staging groups remain coarse, even with the introduction of post-neoadjuvant staging (ie, ypTNM) in TNM 8.^6^ Important characteristics that are readily available and routinely collected (such as circumferential resection margin) are not considered for the sake of simplicity, leading to a range of survival outcomes for patients within the same stage groups. This makes application at the patient level inaccurate.

The delivery of personalized long-term survival estimates after treatment for esophageal cancer is challenging. In addition to informing patients, reliable survival figures would enable the identification of high-risk individuals or groups in whom enhanced surveillance or treatment intensification (with traditional or novel agents such as immunotherapy) could be considered, or conversely patients where de-escalation would be the preferred option.

Prognostic models can address these limitations by combining multiple risk factors, although none have entered widespread use among surgeons or oncologists treating esophageal cancer.^7^,^8^ Models based on machine learning (ML) techniques may produce more accurate predictions than models built using traditional statistical methods (eg, logistic/cox regression).^9^,^10^ In particular, Random Survival Forest (RSF) models have produced promising results^11^–^13^ in various settings, and in esophageal cancer were used to derive the AJCC TNM 7^th^ and 8^th^ edition staging manuals,^5^,^6^ and to quantify the benefits of optimizing treatment.^14^ RSF is a machine-learning method that, when developed to predict survival, builds many decision trees with log-rank test based split points to identify different survival trajectories, with the predicted probability for an individual being derived as the average prediction across all of the trees.

The aim of this study was to derive and validate a prognostic model based on RSF methods for long-term survival after esophagectomy for cancer, and to compare its performance to a model developed using a common statistical approach (Cox regression), using a population-based dataset from England and Wales.

## Methods

### Study Cohort

The study used a linked dataset prepared by the National esophago-Gastric Cancer Audit (NOGCA), a national clinical audit of patients undergoing treatment for cancer of the esophagus or stomach in England and Wales.^15^ The audit was commissioned by the Healthcare Quality Improvement Partnership (HQIP) and funded by NHS England and the Welsh government. Patients were eligible for inclusion in the audit if they had a histological diagnosis of epithelial cancer, with the first patients being registered in April 2012. The audit collects a dataset that covers the care pathway from diagnosis to the end of initial treatment and links these patient records with information from other national health care datasets, including the National Cancer Registration and Analysis Service (NCRAS, see^15^ for more details). Data collection was approved by the Confidentiality Advisory Group under section 251 of the NHS Act 2006.

### Ethics Approval

The study is exempt from UK National Research Ethics Committee approval as it involved secondary analysis of an existing dataset of anonymized data. The National Esophago-gastric (OG) Cancer Audit has approval for processing health care information under Section 251 (reference number: ECC 1–06 (c)/2011) for all National Health Service (NHS) patients diagnosed with OG cancer in England and Wales. Data for this study are based on patient-level information collected by the NHS, as part of the care and support of patients with cancer.

The study cohort included patients diagnosed with adenocar-cinoma or squamous cell carcinoma of either the esophagus or gastroesophageal junction (Siewert I—II) between April 1, 2012 and March 31, 2018 who underwent a planned curative esophagec-tomy. The study excluded patients who died in hospital before discharge, had confirmed metastatic disease on postoperative histology, or had an inadequate lymphadenectomy (<15 lymph nodes),^16^ in whom interpretation of lymph node status would be biased. Supplementary Figures S.1, http://links.lww.com/SLA/C964 and S.2, http://links.lww.com/SLA/C964 details the patient exclusions and assumptions to derive the final sample size (n = 6399).

The primary outcome was overall survival from the date of discharge following surgery. Survival was confirmed by linking the audit records with records from the Office for National Statistics death register. Median duration of follow-up was 41 months (interquartile range 24–59).

### Variable Definition

The audit data contained 41 variables that were routinely measured in clinical practice, were beyond the control of the provider, had > 50% completeness, and were clinically relevant to survival, listed in Table S.1, http://links.lww.com/SLA/C964. The dataset contained patient characteristics, disease information, details of treatment received, postoperative complications, and tissue pathology. Circumferential resection margins were considered involved if there was tumor at <1 mm from the cut edge and longitudinal resection margins were considered involved if tumor was found at the cut edge, in line with Royal College of Pathologists Guidelines.^17^ In patients undergoing neoadjuvant therapy, treatment was specified as “complete” if it was completed as prescribed or “not complete” (due to disease progression, treatment toxicity, technical problems, or patient choice). Malignant esophageal and gastric surgery is centralized in England and Wales and undertaken solely by dedicated teams. We therefore defined annual hospital volume as average number of major upper gastrointestinal resections (esophagectomy/major gastrectomy) per year, in line with NHS commis ioning guidelines.^18^ Staging was conducted using the 8^th^ edition of the AJCC TNM staging manual.

Among the 41 variables considered for inclusion, five had missing values for >5% of patients: completion of neoadjuvant treatment (19.9%), return to theater (15.8%), grade of differentiation (7.0%), cT stage (5.9%), and surgical approach (5.7%). Missing data were assumed to be missing at random and were addressed using multiple imputation by chained equations with 10 imputations.^19^

### Model Development

The study aimed to develop a model using a subset of variables so that data collection would be straightforward, and the model easy to use in clinical settings. To select core variables, we used permutation based Random Forest variable importance (VIMP)^11^ with bootstrapped confidence intervals. Variables with a *P<* 0.01 for VIMP >0 were included in the final model (Table S.2, http://link-s.lww.com/SLA/C964). Pre-treatment histology (ie, adenocarcinoma or squamous cell carcinoma) was also included to improve the face validity of the model. The final model was trained using 14 variables: age, sex, cT, cN, site of tumor, pre-treatment histology, neoadjuvant treatment, completion of neoadjuvant treatment, pT/ypT, number of positive lymph nodes, circumferential and longitudinal margin involvement, grade of differentiation, and presence/absence of surgical complications. The RSF hyperparameters (ie, number of trees, number of variables per tree, and minimum node size) were optimized by grid search. Final predictions were combined across the imputed data.^20^–^22^

A cox regression model was also developed using the same set of variables. Not all relationships between survival and continuous variables were linear, and a square root transformation was adopted for positive lymph nodes, whereas age was included as a restricted cubic spline.

### Assessment of Model Performance

Model performance was quantified by discrimination and calibration. Discrimination was assessed using the time-dependent area under the receiver operator curve (tAUC).^23^ Here this represents the proportion of random pairs of patients (1 alive at time point “t” and 1 dead before this) where the model gives the patient who is alive a higher probability of survival than the patient who is dead. It can be considered analogous to the standard AUC in a binary regression model, extended to survival by weighting of censored patients,^24^ and has advantages over the C-statistic measure of performance.^25^ Assessment of calibration was conducted visually for 5 patient subgroups of increasing risk (ie, patients were grouped by quintiles of predicted risk of mortality at 5 years). In addition, we calculated the integrated brier score.^26^,^27^ A score closer to 0 indicates better accuracy of predictions.

Finally, the relative performance of the 2 models was compared using decision curve analysis.^28^ This method is based on evaluating the “net-benefit” of model predictions across of range of possible decision thresholds that reflect how a patient might weigh the risk of harm associated with a false-positive result (compared with a true positive result). Models with a better performance have a greater net benefit across all thresholds of probability.

Data analysis was conducted in R 3.5.3.^29^ The RSF was trained using the packages Ranger^30^ and RF-SRC.^31^ This study was conducted to comply with the AJCC prognostic model^32^ and TRIPOD^33^ criteria, a compliance checklist is provided in Table S.3, http://links.lww.com/SLA/C964. Complete R code to reproduce the analysis is available on request. More extensive methodology and instructions to perform external validation are provided in the supplementary materials. All performance metrics were validated internally by the 0.632 estimator^34^ in 1000 replications of the bootstrap with replacement.

## Results

**Figure 1:**
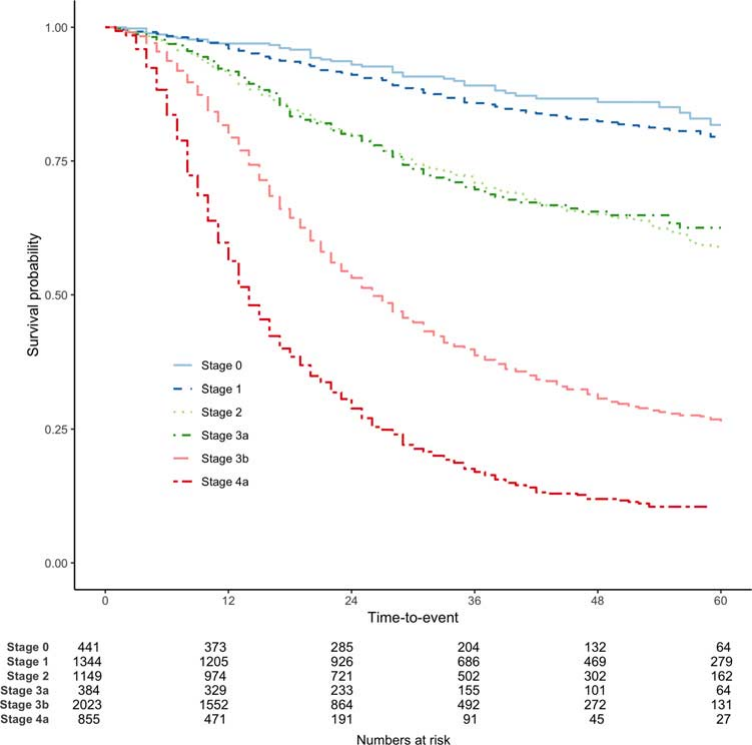
Survival of patients who underwent an esophagectomy between April 2012 and March 2018, stratified by TNM stage.

The study included 6399 patients with esophageal cancer who underwent an esophagectomy between April 2012 and March 2018. Table 1 summarizes the characteristics of patients and their treatment. The median age at diagnosis was 66 years and only 1 in 5 were women. Tumors were predominantly adenocarcinoma (87%) and about 3 in 10 were classified as GEJ-Siewert I-II. There were 2625 recorded deaths, and the median survival was 53 months. Survival at 1, 3, and 5 years was 83.7%, 57.1%, and 47.1% respectively (Fig. 1). Differences in survival stratified by stage according to if patients received neoadjuvant treatment (ie, ypTNM) or surgery alone (ie, pTNM) are shown and discussed in supplementary Figure S.9, http:// links.lww.com/SLA/C964.

**Table 1:** Background Characteristics of Patients Who Underwent An Esophagectomy Between April 2012 and March 2018

Characteristic	N = 6399	Median Survival	Characteristic	N = 6399	Median Survival
Sex			Anastomotic leak		
Male	5045 (78.8)	47	No	5923 (92.6)	54
Female	1354 (21.2)	72	Yes	445 (7.0)	40
Age			Unknown	31 (0.5)	NR
0—40	64 (1.0)	NR	Any complication		
41—50	405 (6.3)	68	No	3810 (59.5)	55
51—60	1397 (21.8)	59	Yes	2558 (40.0)	49
61—70	2615 (40.9)	60	Unknown	31 (0.5)	
71—80	1787 (27.9)	42	Involved longitudinal margin		
81 +	131 (2.0)	29	No	6188 (96.7)	55
Site of tumor			Yes	211 (3.3)	19
Upper/mid esophagus	792 (12.4)	61	Involved circumferential margin		
Lower esophagus	3795 (59.3)	52	No	4617 (72.2)	77
GEJ (S1—2)	1812 (28.3)	53	Yes	1534 (24.0)	21
Histopathology			Unknown	248 (3.9)	57
Adenocarcinoma	5540 (86.6)	51	pT/ypT		
SCC	859 (13.4)	68	T0/is	524 (8.2)	NR
cT			T1	1201 (18.8)	NR
T0/is/1	467 (7.3)	NR	T2	836 (13.1)	NR
T2	1294 (20.2)	67	T3	3549 (55.5)	30
T3	3979 (62.2)	38	T4	289 (4.5)	13
T4	284 (4.4)	36	Lymph nodes examined	26 [15—130]	
Unknown	375 (5.9)	NR	pN/ypN		
cN			N0	2994 (46.8)	NR
N0	2551 (39.9)	76	N1	1414 (22.1)	46
N1	2547 (39.8)	41	N2	1133 (17.7)	22
N2	938 (14.7)	32	N3	858 (13.4)	14
N3	159 (2.5)	28	Grade		
Unknown	204 (3.2)	47	G1 (well)	226 (3.5)	NR
cM			G2 (moderate)	2331 (36.4)	60
M0	6151 (96.1)	54	G3/4 (poor/anaplastic)	2697 (42.1)	38
M1	44 (0.7)	26	GX (unable to determine)	695 (10.9)	66
Unknown	204 (3.2)	47	Unknown	450 (7.0)	72
ASA			NAT		
1	892 (13.9)	64	Chemotherapy	3976 (62.1)	42
2	3745 (58.5)	53	Chemoradiotherapy	450 (7.0)	NR
3	1726 (27.0)	42	None	1973 (30.8)	66
4	36 (0.6)	43	Completion of NAT		
Approach			Completed	2981 (46.6)	51
Open	3357 (52.5)	51	Not completed	282 (4.4)	32
Hybrid	1931 (30.2)	51	Not applicable	1861 (29.1)	67
MIO	748 (11.7)	NR	Unknown	1275 (19.9)	42
Unknown	363 (5.7)	41	Annual hospital volume		
			1–30	504 (7.9)	59
			31–60	3351 (52.4)	55
			>60	2544 (39.8)	48

Data given as absolute number (percentage) and median (Range). MIO indicates minimally invasive esophagectomy; NAT, neoadjuvant treatment; NR, median survival not reached; SCC, squamous cell carcinoma.

A total of 13 variables were identified as important to include in the final model in addition to histological diagnosis. The RSF variable importance measure indicated the number of lymph nodes as the most important single risk factor for worse prognosis followed by pT/ypT stage (see partial dependence plots, supplementary figures S.3/S.4, http://links.lww.com/SLA/C964).

### Model Performance: Internal Validation

**Figure 2:**
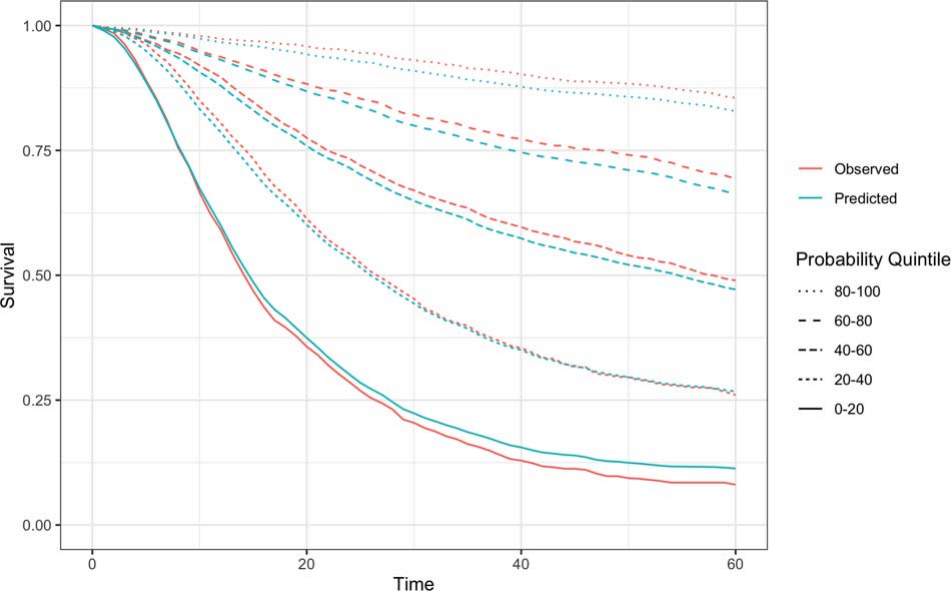
Calibration of predictions from RSF model. Patients grouped into quintiles according to predicted survival at 60 months post-surgery.

The RSF model demonstrated excellent discrimination, with a bootstrapped tAUC at 60 months of 83.9% (95% CI 82.6%–84.9%), which was similar at other time points (Figure S.6, http://links. lww.com/SLA/C964). This was better than the Cox regression model (coefficients of which are given in Table S.4, http://links.lww.com/ SLA/C964), which had a bootstrapped tAUC of 82.3% (95% CI 81.1%–83.3%) and TNM stage alone (tAUC 74.5%). Figure 2 shows the agreement between the RSF model predicted and observed survival times for patients grouped according to quintile of prediction and in both models, calibration was visually good throughout these groups. The integrated brier scores for the RSF model was superior to the cox regression at 0.136 (95% CI 0.134–0.138) and 0.141 (0.139–0.143), respectively. Decision curve analysis also showed a greater net benefit for the RSF over Cox regression model (Figure S.7, http:// links.lww.com/SLA/C964) or using TNM alone (Figure S.8, http:// links.lww.com/SLA/C964).

**Figure 3:**
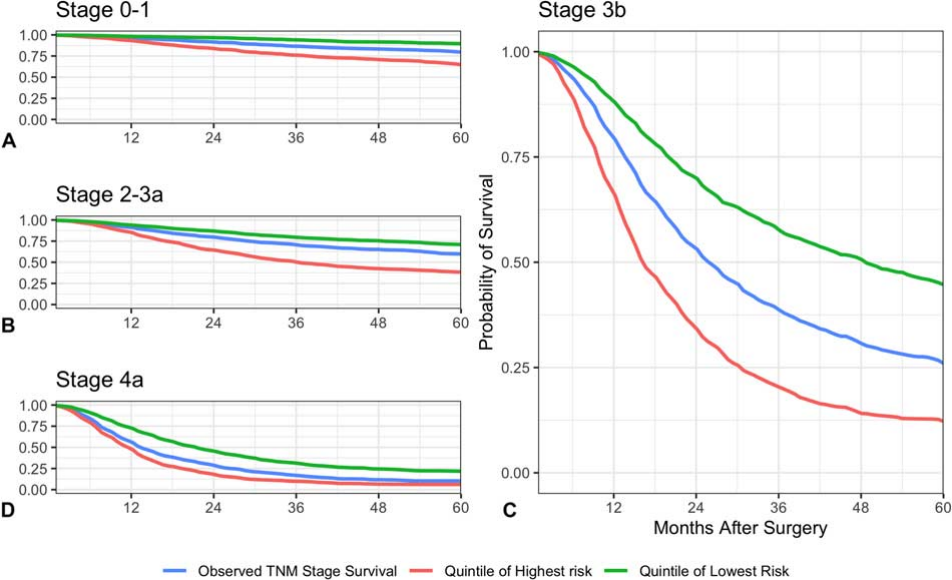
Range of predictions by p/ypTNM stage. A, Stage 0–1. B, Stage 2–3a. C, Stage 3b. D, Stage 4a. Patients were grouped into quintiles by predicted survival at 60 months, with the highest and lowest groups shown.

There were a broad range of predictions yielded even within p/ ypTNM staging groups, with the lowest risk quintile of Stage 3b patients having a predicted 5-year survival of 44.7% compared to 12.2% for the highest risk quintile. Moreover, there is a subgroup of early-stage disease (TNM stage 0—1), who would generally be considered to be cured, who had a relatively poorer survival of only 64.7% at 5 years (Fig. 3) and overlap of quintiles between staging groups.

**Figure 4:**
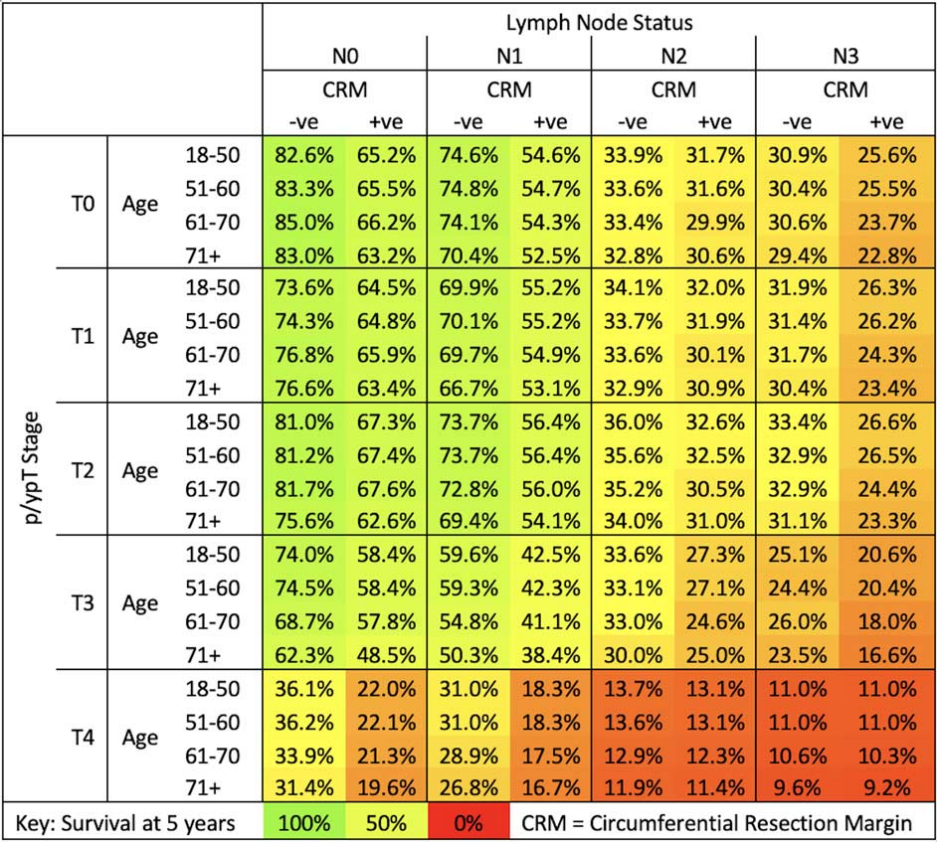
Predicted 5-year survival for a given combination of selected variables. Colors represent differing prognosis, with green more favourable and red less favourable.

Figure 4 gives an overview of mean predicted 5-year survival for combinations of the most important variables (Lymph node status, T-stage, circumferential resection margin involvement, and age at diagnosis). Age at diagnosis is most influential with early stage (T0—2, N0-1) disease; however, its importance diminishes with increasing T/N-stage. Examples of how the model might be used are given in the supplementary materials (Figure S.10, http://link-s.lww.com/SLA/C964)

## Discussion

Accurate predictions of long-term survival following surgery for esophageal cancer may help clinicians and patients. This study has demonstrated that an RSF model can discriminate between patients with different long-term prognoses using a small number of routinely collected variables. The model showed very good calibration and discrimination on internal validation, and exceeded that achieved using Cox regression analysis. The model is applicable to patients who have undergone a planned curative esophagectomy for adenocarcinoma or squamous cell carcinoma of the esophagus, who had an adequate lymphadenectomy and survived to discharge from hospital.

At present, information given to patients after surgery about their long-term survival is limited and is largely based on TNM staging. This can mean the information provided to patients can be vague, such as “50% survive to 5 years.” Decisions on whom to offer adjuvant treatment or consider for entry into trials may involve more criteria than TNM staging, but the relationship between these criteria and survival may be uncertain.

The model described here provides a more precise prediction of prognosis for an individual patient than TNM staging alone, and this will be valuable in postoperative discussions with patients. This increased accuracy has several benefits. In a research setting, it is key for establishing the efficacy of treatment. In clinical practice, it supports selecting the right patients for the right treatments, particularly with the emergence of novel therapies (eg, Immune checkpoint inhibitors^35^). Further research on how best to communicate predicted survival to patients is required; even in early-stage disease, desire for detail of prognosis is highly variable,^36^ and the effective use of decision aids is challenging.^37^

The model compares favorably to those published previously. Cox proportional hazard models using a variety of predictors have reported C-index/tAUCs between 0.61 and 0.70.^38^—^40^ In comparison the C-index of our model was 0.76 (0.75—0.78) and the 5-year tAUC 0.84 (0.83—0.85). It also is more broadly applicable and includes patients with all modalities of neoadjuvant treatment.

The variables found to be most influential are consistent with clinical experience and the findings from other studies, with lymph node involvement being widely recognized as the most influential determinant of long-term survival in esophageal cancer. Both clinical T stage and clinical N stage were found to be important independent of their pathological equivalents. There is some logic to support including both clinical and pathological variables, in that changing variables within patients may indicate the impact of neoadjuvant treatment (although this is limited by the relatively decreased accuracy of clinical staging). This is supported by recent studies which have shown that downstaging after neoadjuvant treatment improves absolute survival independent of the ypTNM stage.^41^ Completion of neoadjuvant treatment was included which is biologically sensible, and important in the context of the increasing use of potentially more toxic regimens such as FLOT.^4^

The main strength of this study is the large sample size from a national population. The case ascertainment of esophagectomies exceeds 90% in the national audit, and the dataset was representative of patients within England and Wales who underwent curative surgery. Another strength is the linkage of audit records with ONS mortality data which enabled complete follow-up.

There are a number of limitations to the approaches taken in this study. Despite being more accurate than TNM staging at the individual patient level in the postoperative setting, no attempt has been made to develop a pre-treatment predictive model and cTNM remains the criterion standard in this domain. The NOGCA lacks several data items known to influence survival such as tumor regression grade^42^ and lymphovascular invasion.^43^ Additionally length of tumor^44^ and BMI^45^ could be considered, but were only available in recent years and therefore had too many missing values. There was also no clear information on what adjuvant treatment this patient cohort had received in addition to their neoadjuvant/ perioperative treatment.

Involvement of circumferential margin was defined according to Royal College of Pathologists criteria, that is, <1 mm from cut edge is involved. Throughout much of the rest of the world the American College of Pathologist guidelines are used,^46^ that is, involved if tumor at cut edge. There is considerable debate about the most appropriate measure,^47^ and this model requires validation if it is to be used with this definition. It was not possible to use T stage subdivided into “a” or “b” because not all patients were recorded with this information. Consequently, the analysis used the base T stage only. Patients treated solely with endoscopic techniques (muco-sal resection or submucosal dissection) who did not require surgery were also excluded and it is not appropriate to use the model in this patient group.

The Esophageal Complications Consensus Group—ECCG^48^ has recently specified and defined a core set of complications for esophagectomy which has been adopted worldwide.^49^ The NOGCA relies on reporting from local cancer centers and pragmatically uses a limited set of complications with broader definitions. In this study, the reported rate of complications was 40.0%, which is significantly less than the figures from the ECCG data (59%). This is likely to reflect the varying definitions and under reporting rather than a truly lower rate, which may explain the low overall importance of complications and absence of specific complications (eg, anastomotic leak) in the model. The ECCG classification of complications has recently been adopted into the national Cancer Outcomes and Services Dataset used in cancer registration within England, so more accurate complication data will be available in future iterations of the model.

## Conclusions

Using a large, nation-wide, contemporaneous clinical dataset, this study has demonstrated the ability of a Random Survival Forest model to provide accurate predictions of long-term survival after surgery for esophageal cancer. A key benefit of the model is its performance in identifying patients with the same disease stage who have diverging 5-year survival. For example within Stage 3b, the largest group with 2023 patients, the model identifies a low-risk quintile of patients with a predicted 5-year survival >3 times the highest risk quintile (44.7% vs 12.2%). These groups will likely benefit from different post-operative monitoring and/or treatment strategies. A similar pattern is seen with stage 4a disease (21.7% vs 6.1% 5-year survival), suggesting that there is a subgroup even in the most advanced (nonmetastatic disease) who might be well served by targeted intervention.

The RSF model described in this article is available at https:// uoscancer.shinyapps.io/AugisSurv/ and could be a valuable prognostication tool for patients, surgeons, and oncologists. In the future, it may also be useful to guide adjuvant treatment. External validation of this tool in other health care systems would be of benefit to confirm its performance.

## Supplementary Material
