# Supplementary material for: The AUGIS Survival Predictor: Prediction of Long-Term and Conditional Survival After Esophagectomy Using Random Survival Forests
Source: Ann Surg. 2023 Jan 10;277(2):267–74. doi: 10.1097/SLA.0000000000004794 (PMC9831040; doi:10.1097/SLA.0000000000004794)
Supplement: Supplementary file 1 [file sla-277-0267-s001.pdf]

**Supplemental Digital Content 1.** Complications

| |
|---|
| Superficial Incisional SSI |
| Urinary Tract Infection (UTI) - CAUTI |
| Stroke/CVA |
| Cardiac Arrest Intraop |
| Cardiac Arrest req. CPR - Postop |
| Myocardial Infarction – Intraop |
| Myocardial Infarction - Postop |
| Cardiac Dysrhythmias |
| Transfusions w/in first 72 hrs postop |
| Deep Vein Thrombosis req. Therapy |
| Sepsis |
| Deep Incisional SSI |
| Severe Sepsis/Septic Shock |
| C-difficile |
| CLABSI |
| Hysterectomy - Bleeding Complications |
| Hysterectomy - Ileus/Small Bowel Obstruction |
| Hysterectomy - Bowel Injury |
| Hysterectomy - Ureteral Obstruction |
| Hysterectomy - Bladder Injury |
| Hysterectomy - Fistula |
| Hysterectomy - Vaginal Cuff Dehiscence |
| Organ/Space SSI |
| Hysterectomy - Vaginal Cuff Cellulitis |
| Anastomotic Leak |
| Septic Shock |
| Pneumonia |
| Unplanned Intubation – Postop |
| Pulmonary Embolism |
| Acute Kidney Injury |
| Urinary Tract Infection (UTI) - Non-CAUTI |
